# Novel Y-chromosome Short Tandem Repeat Variants Detected Through the Use of Massively Parallel Sequencing

**DOI:** 10.1016/j.gpb.2015.08.001

**Published:** 2015-09-21

**Authors:** David H. Warshauer, Jennifer D. Churchill, Nicole Novroski, Jonathan L. King, Bruce Budowle

**Affiliations:** 1 Institute of Applied Genetics, Department of Molecular and Medical Genetics, University of North Texas Health Science Center, Fort Worth, TX 76107, USA; 2 Center of Excellence in Genomic Medicine Research (CEGMR), King Abdulaziz University, Jeddah 21589, Saudi Arabia

**Keywords:** Y-STR, Sequence polymorphism, Allele variants, Massively parallel sequencing, Nextera, STRait Razor

## Abstract

**Massively parallel sequencing** (MPS) technology is capable of determining the sizes of short tandem repeat (STR) alleles as well as their individual nucleotide sequences. Thus, single nucleotide polymorphisms (SNPs) within the repeat regions of STRs and variations in the pattern of repeat units in a given repeat motif can be used to differentiate alleles of the same length. In this study, MPS was used to sequence 28 forensically-relevant Y-chromosome STRs in a set of 41 DNA samples from the 3 major U.S. population groups (African Americans, Caucasians, and Hispanics). The resulting sequence data, which were analyzed with **STRait Razor** v2.0, revealed 37 unique allele sequence variants that have not been previously reported. Of these, 19 sequences were variations of documented sequences resulting from the presence of intra-repeat SNPs or alternative repeat unit patterns. Despite a limited sampling, two of the most frequently-observed variants were found only in African American samples. The remaining 18 variants represented allele sequences for which there were no published data with which to compare. These findings illustrate the great potential of MPS with regard to increasing the resolving power of STR typing and emphasize the need for sample population characterization of STR alleles.

## Introduction

Short tandem repeat (STR) markers located on the Y-chromosome (Y-STRs) are extremely useful because of a lack of recombination. Barring mutation, all paternally-related males share the same Y-STR haplotype. As a result, Y-STRs are used in genealogical and evolutionary studies, and forensic genetics casework including paternity testing to determine the biological father of a particular male child, missing persons cases where the Y-STR haplotype can serve as an extended reference profile for a given paternal lineage, and analyses of mixture evidence where there is substantially more female DNA than male DNA. Indeed, the variety of uses for Y-STR markers has made them the object of extensive research and application within the scientific community.

Given the value of STR markers in identity testing, efforts are underway to increase the power of discrimination associated with their respective typing and analysis methods. Primarily, an increase in power of discrimination has been accomplished through the introduction of new, highly-polymorphic STRs and by developing larger multiplex panels [1–4]. Discrimination power also may be increased by further characterization beyond nominal length of the alleles at extant loci. STR alleles are typically characterized by the number of units in their repeat motifs, a distinction commonly determined by size separation by capillary electrophoresis (CE). However, other detection methods, such as Sanger sequencing and mass spectrometry, have been used to determine both the size and the nucleotide composition of STR alleles [5,6]. The emergence of massively parallel sequencing (MPS) technologies improved upon this principle by allowing for the detection of a substantially-larger amount of genetic sequence information with a higher throughput, lower cost, and greater ease-of-use than previous methods. Studies involving each of these approaches have resulted in the detection of intra-repeat single nucleotide polymorphisms (SNPs) and novel repeat motif variants, which allow for a greater level of distinction than that of traditional CE methods [5–10]. For instance, two individuals with the same nominal allele(s) (based on length) at a certain locus potentially may be distinguished by MPS if the nucleotide sequence of the allele differs between them. This level of resolution may prove invaluable in the deconvolution of genetic mixtures and also could provide information about population-specific alleles for evolutionary studies.

In this proof-of-principle study, MPS was used to determine the repeat sequences of 28 forensically-relevant Y-STRs across a dataset of three major US populations (*n* = 41): Caucasians (CAU), Hispanics (HIS), and African Americans (AFA). These sequence data revealed several intra-repeat SNPs and allelic variants that have not been documented previously. The novel variants described herein are indicative of the potential of MPS with regard to identifying additional genetic diversity of Y-STRs and support that more in depth population studies are warranted.

## Results

Since nanogram and subnanogram quantities of input DNA can be typed by MPS, PCR enrichment has become the method of choice for studies involving forensic applications. However, this study employed a capture enrichment approach. The TruSeq library preparation chemistry was selected initially, because no PCR amplification is required. Therefore, primer binding site mismatch issues would not impact multiplex design or the amplification success. It was hypothesized that a dense probe design would increase capture efficiency of the target loci. In addition, PCR-generated errors would be reduced, thus minimizing potential artifacts. Lastly, laying a foundation of sequence data with an alternate enrichment system could be useful when full validation studies are undertaken.

### Sequencing coverage

All 28 Y-STR loci were detected with the approach described herein. The coverage ranged from 0 to 1493 ×, with a mean coverage of 9−387 ×. The lowest performing markers were DYS448 (mean 9 ×), DYS449 (mean 33 ×), DYS518 (mean 34 ×), DYS389II (mean 37 ×), and DYS505 (mean 38 ×); while the highest were DYS643 (mean 322 ×), DYS391 (mean 333 ×), and DYS438 (mean 387 ×).

### Sequence variants

A total of 37 unique Y-STR allele sequences that have not been previously published were detected across the 41 samples used in this study. These sequences may be divided into 2 categories: nominal allele variant sequences and novel allele sequences. For the purposes of this study, a nominal allele variant sequence is defined as any allele sequence that differs from the previously-documented sequence(s) for that particular allele, whereas a novel allele sequence refers to the sequence detected for an allele that has no previously published sequence data with which to compare.

#### Nominal allele variants

Of the 37 previously-undocumented allele sequences that were detected, 19 were classified as nominal allele variant sequences. These nominal variants were found in loci DYS389I/II, DYS390, DYS393, DYS481, DYS518, and DYS635, and have been further characterized as either SNP variants or repeat pattern variants (RPVs) (Table 1). Allele sequence variation may be introduced via strand slippage or one or more point variations within the repeat region. In this study, nominal variant sequences were classified as SNP variants if they displayed a repeat motif that differs from the commonly-described motif, an occurrence indicative of point substitution. RPVs are defined as allele sequences that differ from published data with regard to repeat unit arrangement, but are consistent with the reported repeat motif. Such variations may be due to strand slippage or the presence of intra-repeat SNPs, but definitive conclusions cannot be made without additional data. To illustrate the differences between these two types of variants, consider a locus with a reported repeat motif of [TCTA]*_n_*[TCTG]*_p_* (where *n* and *p* represent the number of repeats). If a “17” allele was detected with a repeat motif of [TCTA]_5_**[TATA]_1_**[TCTG]_11_, this nominal allele variant sequence would likely be due to the presence of a C/A SNP in the first “TCTA” repeat unit. Since such a change results in a “TATA” repeat unit that is inconsistent with the reported repeat motif, this sequence would be classified as a SNP variant. However, if another nominal variant was detected for this allele with a repeat motif of [TCTA]_6_[TCTG]_11_, it would be labeled a RPV, as the structure remains consistent with the reported repeat motif but displays a pattern of repeat units that has not been previously documented.

The unique sequence detected for allele “9” at locus DYS389I is particularly interesting, as it completely lacks the “TCTG” repeat unit found in the locus’ repeat motif, [TCTG]*_q_*[TCTA]*_r_* (*q* and *r* represent the number of a particular repeat within STR). Instead, the variant allele, observed in only 1 Caucasian sample, consists entirely of “TCTA” repeats. The published sequence for this allele consists of 3 “TCTG” and 6 “TCTA” repeat units. Since the “TCTG” repeat unit, as defined in the reported repeat motif, is variable, its absence was not considered an inconsistency with regard to the motif, and this novel sequence is therefore deemed a RPV. In total, only three of the 19 Y-STR nominal variants were SNP variants. At locus DYS393, an A/C SNP in the variable “AGAT” repeat unit produced a leading “CGAT” unit in allele “13”. Additionally, a T/G SNP in the variable “CTT” repeat unit of alleles “25” and “26” at locus DYS481 resulted in the presence of a leading “CTG” repeat in both of these alleles. This SNP variation was previously characterized by Geppert and colleagues [7] in allele “21”, which also was detected in the current study.

In addition to the effects of SNPs, the nominal allele sequences detected in this study highlight a high degree of allele variability at certain loci due to RPV. Locus DYS518, for instance, displayed multiple variants for all but one allele, some of which were previously characterized by D’Amato and colleagues [8]. These variations are due to differences in the numbers of the two variable “AAAG” repeat units at this locus. Finally, one of the detected sequence variations for the “23” allele at locus DYS635 (GATA-C4) is particularly interesting. This locus exhibits a wide range of allele variation due to the presence or absence of two “TGTA” repeats among the trailing “TCTA” repeat units, an occurrence that has been described previously in STRBase (http://www.cstl.nist.gov/strbase/ystr_fact.htm and http://www.cstl.nist.gov/strbase/srm2395.htm) and by Oloffson and colleagues [11]. However, the “23” allele detected in this study contained three “TGTA” repeats, resulting in a sequence variant that has not been characterized until now.

The majority of these nominal allele sequence variants displayed a low frequency of occurrence across the dataset, with 16 of the 19 allele variants detected in only one single sample each. However, the previously-described RPVs observed for allele “30” at locus DYS389II and for allele “21” at locus DYS390 were detected in 7 and 8 samples, respectively. Interestingly, these variants occurred exclusively in African American samples, indicating that these alternative allele sequences may be population-specific and also may reflect the known greater genetic diversity in the African population. For the most part, other frequently-observed sequence variants appeared to be fairly evenly parsed among at least two populations.

The majority of the allele sequences detected at the 28 targeted loci were consistent with previously-published sequences (data not shown). Noteworthy examples include the microvariant alleles “13.2” and “17.2” at loci DYS385 and DYS458, respectively, both of which have been previously characterized by Myers and colleagues [12,13]. At these loci, the microvariant alleles occur as a result of a “GA” deletion in the variable “GAAA” repeat unit.

#### Novel allele variants

In addition to the large number of observed sequences that have been documented previously, a total of 18 novel allele sequences were detected across the 41 samples analyzed (Table 2). The number of samples in which these novel sequences were observed ranged from 1 to 13, although many occurred relatively infrequently across the dataset. The novel allele sequences included two SNP variants. At locus DYS570, a T/C SNP in allele “23” resulted in a sequence change from [TTTC]_23_ to [TTTC]_5_**[TCTC]_1_**[TTTC]_17_. Another T/C SNP, observed in allele “35” at locus DYS612, changed the repeat sequence from [CCT]_5_[CTT]_1_[TCT]_4_[CCT]_1_[TCT]_30_ to [CCT]_5_[CTT]_1_[TCT]_4_[CCT]_1_[TCT]_17_**[CCT]_1_**[TCT]_12_. The remaining novel sequences, such as those detected at locus DYS635, were consistent with the described repeat motifs of their respective alleles.

### Y-STR haplogroup assignment

Lastly, haplogroup assignments were made for each Y-STR profile based on the number of repeats of each locus of a haplotype (Table S1). While there are sequences that are associated with specific haplogroups, the sample size is too small to make any population inferences. The haplogroups are provided for each of the reported allele sequences as these may prove useful for future population studies.

## Conclusions

The unique allele sequence variants detected in this study have been presented to demonstrate that additional characterization of Y-STR alleles is feasible by sequencing. The results also provide some insight into the mechanism of allele variant occurrence. While SNP variants were detected, the majority of novel sequences consisted of repeat pattern variants. Although the exact mechanism of mutation for the repeat pattern variants observed in this study cannot be definitively concluded, it should be noted that the majority of STR variation has been attributed to strand slippage [14–16]. Therefore, even if a single point mutation event may seem to be the most parsimonious explanation for a repeat pattern variant, a two-step strand slippage event may be more probable. Such concepts must be taken into account when characterizing these novel variants. Regardless of their mechanism of introduction, the presence of intra-repeat SNPs and repeat pattern variations in Y-STR alleles may aid in the differentiation of males sharing the same nominal alleles, and perhaps even paternally-related males, in forensic casework samples. Given its ability to detect both the length of STR alleles and their individual nucleotide sequences, MPS technology offers more resolution with regard to STRs than traditional length-based detection methods, such as CE. CE would yield the size of an amplicon, *i.e*., equivalent of repeat length, which can be ascertained from sequence data simply by counting the number of nucleotides within the repeat region. To date, the vast majority of STR nominal length results have been the same among different platforms and systems (data not shown). While the dataset used in this study was relatively small, the large number of observed novel allele sequence variants highlights the need for characterization of Y-STR alleles in larger sample populations.

## Materials and methods

### Samples and extraction

Following the University of North Texas Health Science Center Institutional Review Board approval, DNA was extracted from whole blood samples from 41 unrelated anonymized individuals, consisting of 12 Caucasian males, 16 Hispanic males, and 13 African American males. These populations were selected because they represent the three major populations in the geographic region. Extraction was performed using the Qiagen QIAamp DNA Mini Kit (Qiagen, Hilden, Germany), according to the manufacturer’s suggested protocol.

### Panel design

The Nextera Rapid Capture Custom Enrichment panel employed in this study was designed using the Illumina Design_Studio sequencing assay design tool. Nextera Rapid Capture chemistry (Illumina, San Diego, CA) is based on enzymatic tagmentation and probe-based capture enrichment. Custom oligonucleotide probes were designed to detect the following 28 forensically-significant Y-STRs: DYS19, DYS385, DYS389I/II, DYS390, DYS391, DYS392, DYS393, DYS437, DYS438, DYS439, DYS448, DYS449, DYS456, DYS458, DYS460, DYS481, DYS505, DYS518, DYS522, DYS533, DYS549, DYS570, DYS576, DYS612, DYS635, DYS643, and GATA-H4. Multiple probes were used for each Y-STR to improve enrichment efficiency.

Probes (80 bases in length) for the Nextera Rapid Capture Custom Enrichment Kit were designed using Design Studio (Illumina), a freely-available software. The STRs were tabulated including details regarding chromosomal positioning, target selection (full region), probe density requirements (due to the alignment-specific requirements of STRs, density of these markers was set at ‘ADJACENT’), and marker information. Marker data then were uploaded to Design Studio v1.5 and probes were generated under the default conditions, with the hg19 human genome assembly (http://hgdownload.cse.ucsc.edu/goldenpath/hg19/chromosomes/) used for probe reference.

### Quantification and normalization

50 ng of genomic DNA was used as the input amount for typing. To bring the 41 extracted DNA samples to the desired input concentration of 5 ng/μl for the Nextera Rapid Capture Custom Enrichment protocol, the quantity of each DNA sample was determined using the Qubit fluorometric quantification method (Thermo Fisher Scientific, Waltham, MA) and normalized to 10 ng/μl with a 10 mM Tris–HCl solution at pH 8.5. The samples then were quantified again and normalized in the same manner to a final concentration of 5 ng/μl, to ensure that the proper amount of genomic DNA would be used for the library preparation process.

### Library preparation

As required by the Nextera Rapid Capture Custom Enrichment protocol, 10 μl of each normalized sample was used for library preparation, for a total of 50 ng of genomic DNA per sample. The samples first underwent tagmentation by the Nextera transposome, whereby the samples are enzymatically cleaved and bound to sequencing adapters [17], at 58 °C in an Applied Biosystems GeneAmp PCR System 9700 thermal cycler (Thermo Fisher Scientific, South San Francisco, CA). The tagmented samples then were purified via two magnetic bead-based 80% ethanol washes, and the fragment sizes of a small subset of these samples were analyzed using the Agilent 2200 TapeStation (Agilent Technologies, Santa Clara, CA) to ensure that the tagmentation process was successful. Dual Nextera sequencing indices then were attached to each of the tagmented samples by amplification in an Eppendorf Mastercycler Pro S thermal cycler (Eppendorf, Hamburg, Germany), using the following parameters: 72 °C for 3 min, 98 °C for 30 s, 10 cycles of 98 °C for 10 s, 60 °C for 30 s, and 72 °C for 30 s, a final extension at 72 °C for 5 min, and a final hold at 10 °C. Following bead-based amplification cleanup with 80% ethanol, each indexed sample was quantified using the Qubit platform. The samples then were normalized and pooled for sequencing, 12 at a time, such that each library contained 500 ng of each uniquely-indexed sample, for a total of 6000 ng of genomic DNA per pool. It should be noted that all libraries consisted of 12 samples. The pooled libraries were hybridized once to the custom oligonucleotide probes in an Eppendorf Mastercycler Pro S thermal cycler, using the following parameters: 95 °C for 10 min, 18 cycles of 1-min incubation, starting at 94 °C, then decreasing 2 °C per cycle, and a final hold at 58 °C for approximately 12 h. A streptavidin bead-based cleanup step was performed wherein the libraries were washed twice for 30 min with an enrichment wash solution at 50 °C. A second hybridization then was performed, using the same thermal cycling parameters, except that the final hold at 58 °C was extended to approximately 20 h. Following a second heated streptavidin bead-based cleanup, the libraries underwent two additional magnetic bead-based washes with 80% ethanol. The libraries then were enriched through amplification in an Eppendorf Mastercycler Pro S thermal cycler, using the following parameters: 98 °C for 30 s, 12 cycles of 98 °C for 10 s, 60 °C for 30 s, and 72 °C for 30 s, a final extension at 72 °C for 5 min, and a final hold at 10 °C. A final magnetic bead-based cleanup procedure was performed, consisting of 2 washes with 80% ethanol, and the libraries were quantified using the Qubit platform. Following quantification, each library was analyzed on the Agilent 2200 TapeStation to determine the average size of the enriched fragments.

### MiSeq sequencing and data analysis

The concentration and size, in base pairs, of the Nextera Rapid Capture Custom Enrichment libraries were used to determine their molarity. To prepare for sequencing on the MiSeq (Illumina), each library was normalized to 2 nM using a solution of 10 mM Tris–HCl buffer (pH 8.5) with 0.1% Tween 20. Illumina’s library preparation guidelines for the MiSeq were followed, and the concentration of each library was adjusted to 12 pM using chilled HT1 hybridization buffer. Paired-end sequencing was performed using the MiSeq Reagent Kit v2, with a read length of 250 bases.

**Figure 1:**
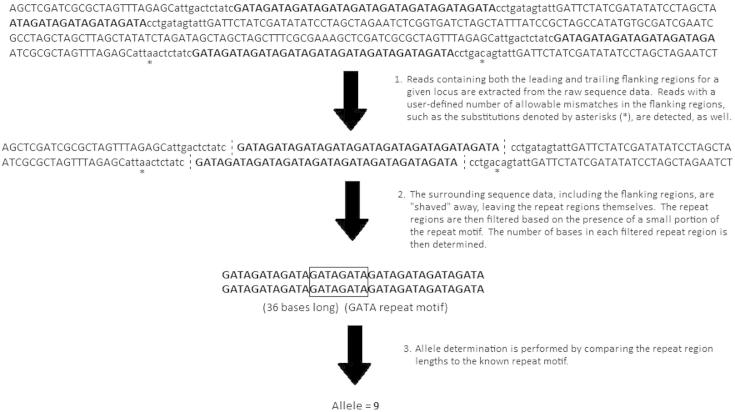
**STRait Razor algorithm for detection of STR alleles** The repeat region is shown in bold, capitalized font, while the flanking regions are shown in plain, lowercase font. Surrounding sequences are shown in plain, capitalized font.

STRait Razor v2.0 [18] was used to analyze the FASTQ files produced by MiSeq for each sample. STRait Razor’s STR allele detection method allows it to genotype alleles found in raw sequence data based on their length, while retaining their individual nucleotide sequences (Figure 1). For the purposes of the current study, a minimum coverage threshold of 5× was used for STR allele determination. The sequence data produced by STRait Razor for each of the targeted Y-STRs across all samples were analyzed using STRait Razor Sequence Analysis [18], and the unique sequences associated with each allele were identified with the STRait Razor Unique Sequences Compiler (https://www.unthsc.edu/graduate-school-of-biomedical-sciences/molecular-and-medical-genetics/laboratory-faculty-and-staff/strait-razor/). These unique sequences then were compared to the known sequences for those alleles that have been previously published in STRBase (http://www.cstl.nist.gov/strbase/srm2395.htm and http://www.cstl.nist.gov/strbase/srm2395.htm) and the literature [7,8,11–13,19].

Y-STR haplogroups were predicted from the repeat lengths (*i.e.*, operationally-defined number of repeats) of the STR alleles comprising the haplotype using Haplogroup Predictor (http://www.hprg.com/hapest5/).

## Authors’ contributions

DHW designed, carried out and analyzed the data of the study and wrote the manuscript; JDC and NN contributed to sample analysis and writing of the manuscript; JLK contributed to study design, data analysis and writing of the manuscript; BB designed the study and contributed to writing and review of the data and manuscript. All authors read and approved the final manuscript.

## Competing interests

The authors have declared no competing interests.

## Figures and Tables

**Table 1:** Nominal allele sequence variants that differ from the published sequences

**Locus**	**Reference repeat motif**	**Allele ID**	**Observed repeat motif**	**Coverage (×)**	**Counts in each population**			**Variant type**	**Associated haplogroups**
**AFA**	**CAU**	**HIS**
DYS389I	[TCTG]_3_[TCTA]*_n_*	9	[TCTA]_9_	60	0	1	0	RPV	R1b
DYS389II	[TCTG]*_n_*[TCTA]*_p_*N48[TCTG]_3_[TCTA]*_q_*	29	[TCTG]_6_[TCTA]_10_N_48_[TCTG]_3_[TCTA]_10_	25	0	0	1	RPV	E1b1b
[TCTG]_6_[TCTA]_11_N_48_[TCTG]_3_[TCTA]_9_	6	1	0	0	RPV	E1b1a
30	[TCTG]_6_[TCTA]_11_N_48_[TCTG]_3_[TCTA]_10_	5–29	1	0	0	RPV	E1b1a
31	[TCTG]_6_[TCTA]_11_N_48_[TCTG]_3_[TCTA]_11_	8	0	1	0	RPV	E1b1a
32	[TCTG]_6_[TCTA]_13_N_48_[TCTG]_3_[TCTA]_10_	6	1	0	0	RPV	E1b1b
DYS390	[TCTG]_8_[TCTA]*_n_*[TCTG]_1_[TCTA]_4_	21	[TCTG]_8_[TCTA]_8_[TCTG]_1_[TCTA]_4_	18–188	1	0	0	RPV	E1b1a
[TCTG]_8_[TCTA]_9_[TCTG]_1_[TCTA]_3_	72	1	0	0	RPV	E1b1b
DYS393	[AGAT]*_n_*	13	[CGAT]_1_[AGAT]_12_	59	0	1	0	A/C SNP	R1a
DYS481	[CTT]*_n_*	25	[CTG]_1_[CTT]_24_	413	0	1	0	T/G SNP	I2a
26	[CTG]_1_[CTT]_25_	211	0	1	0	T/G SNP	E1b1a
DYS518	[AAAG]_3_[GAAG]_1_[AAAG]*_n_*[GGAG]_1_[AAAG]_4_N_6_[AAAG]*_p_*	36	[AAAG]_3_[GAAG]_1_[AAAG]_14_[GGAG]_1_[AAAG]_4_N_6_[AAAG]_13_	31	0	0	1	RPV	G2a
37	[AAAG]_3_[GAAG]_1_[AAAG]_16_[GGAG]_1_[AAAG]_4_N_6_[AAAG]_12_	13	0	1	0	RPV	R1b
38	[AAAG]_3_[GAAG]_1_[AAAG]_14_[GGAG]_1_[AAAG]_4_N_6_[AAAG]_15_	44	0	0	1	RPV	J2a
[AAAG]_3_[GAAG]_1_[AAAG]_15_[GGAG]_1_[AAAG]_4_N_6_[AAAG]_14_	10–68	2	2	1	RPV	E1b1a, I2a, J2b, R1b
39	[AAAG]_3_[GAAG]_1_[AAAG]_18_[GGAG]_1_[AAAG]_4_N_6_[AAAG]_12_	26	0	0	1	RPV	I2b
40	[AAAG]_3_[GAAG]_1_[AAAG]_18_[GGAG]_1_[AAAG]_4_N_6_[AAAG]_13_	22	1	0	0	RPV	E1b1a
41	[AAAG]_3_[GAAG]_1_[AAAG]_16_[GGAG]_1_[AAAG]_4_N_6_[AAAG]_16_	22	0	1	0	RPV	R1a
DYS635	[TCTA]_4_[TGTA]_2_[TCTA]_2_[TGTA]_2_[TCTA]_2_[TGTA]*_n_*[TCTA]*_p_*	23	[TCTA]_4_[TGTA]_2_[TCTA]_2_[TGTA]_2_[TCTA]_2_[TGTA]_3_[TCTA]_8_	247	0	0	1	RPV	R1b

*Note: n*, *p*, and *q* represent number of individual repeats per short tandem repeat unit. AFA, African American; CAU, Caucasian; HIS, Hispanic; RPV, repeat pattern variant. Reference motifs are based on sequences provided in STRBase (http://www.cstl.nist.gov/strbase/ystr_fact.htm) and those published by D’Amato and colleagues [8]. SNP in the observed repeat motif is underlined.

**Table 2:** Novel allele sequence variants

**Locus**	**Reference repeat motif**	**Allele ID**	**Observed repeat motif**	**Coverage (×)**	**Counts in each population**			**Variant type**	**Associated haplogroups**
**AFA**	**CAU**	**HIS**
DYS449	[TTTC]*_n_*N_50_[TTTC]*_p_*	25	[TTTC]_11_N_50_[TTTC]_14_	10	0	0	1	RPV	J1
DYS505	[TCCT]*_n_*	11	[TCCT]_11_	28–55	1	2	5	RPV	E1b1b, G2a, I1, O/Q, R1b
14	[TCCT]_14_	24	1	0	0	RPV	E1b1a
DYS533	[ATCT]*_n_*	9	[ATCT]_9_	113	0	0	1	RPV	G2a
11	[ATCT]_11_	8–629	4	5	4	RPV	E1b1a, E1b1b, I1, J2a, O/Q, R1b
13	[ATCT]_13_	83–458	1	1	2	RPV	R1b
14	[ATCT]_14_	129	0	1	0	RPV	R1b
DYS549	[GATA]*_n_*	10	[GATA]_10_	362–402	1	1	0	RPV	E1b1a, I2a
11	[GATA]_11_	15–390	5	0	1	RPV	E1b1a, E1b1b
DYS570	[TTTC]*_n_*	23	[TTTC]_5_[TCTC]_1_[TTTC]_17_	192	0	0	1	T/C SNP	E1b1b
DYS576	[AAAG]*_n_*	13	[AAAG]_13_	360	1	0	0	RPV	E1b1a
22	[AAAG]_22_	149	0	0	1	RPV	R1b
DYS612	[CCT]_5_[CTT]_1_[TCT]_4_[CCT]_1_[TCT]*_n_*	35	[CCT]_5_[CTT]_1_[TCT]_4_[CCT]_1_[TCT]_17_[CCT]_1_[TCT]_12_	122	0	0	1	T/C SNP	J2b
DYS635	[TCTA]_4_[TGTA]_2_[TCTA]_2_[TGTA]_2_[TCTA]_2_[TGTA]*_n_*[TCTA]*_p_*	24	[TCTA]_4_[TGTA]_2_[TCTA]_2_[TGTA]_2_[TCTA]_2_[TGTA]_2_[TCTA]_10_	9	1	0	0	RPV	R1b
25	[TCTA]_4_[TGTA]_2_[TCTA]_2_[TGTA]_2_[TCTA]_2_[TGTA]_2_[TCTA]_11_	23–28	0	1	0	RPV	R1b
26	[TCTA]_4_[TGTA]_2_[TCTA]_2_[TGTA]_2_[TCTA]_2_[TGTA]_2_[TCTA]_12_	13	1	0	0	RPV	R1b
DYS643	[CTTTT]*_n_*	8	[CTTTT]_8_	395	0	0	1	RPV	J2a
14	[CTTTT]_14_	34	1	0	0	RPV	E1b1a

*Note: n* and *p* represent number of individual repeats per short tandem repeat unit. AFA, African American; CAU, Caucasian; HIS, Hispanic; RPV, repeat pattern variant. Reference motifs are based on sequences provided in STRBase (http://www.cstl.nist.gov/strbase/ystr_fact.htm) and those published by D’Amato and colleagues [8] and Butler and colleagues [19]. SNP in the observed repeat motif is underlined.

## Supplementary material

Supplementary material 1 **Y-STR haplotypes with haplogroup assignment and haplogroup probability value**.

## References

[1] Flores S., Sun J., King J., Budowle B. (2014). Internal validation of the GlobalFiler Express PCR Amplification Kit for the direct amplification of reference DNA samples on a high-throughput automated workflow. Forensic Sci Int Genet.

[2] Oostdik K., Lenz K., Nye J., Schelling K., Yet D., Bruski S. (2014). Developmental validation of the PowerPlex Fusion System for analysis of casework and reference samples: a 24-locus multiplex for new database standards. Forensic Sci Int Genet.

[3] Mulero J.J., Chang C.W., Calandro L.M., Green R.L., Li Y., Johnson C.L. (2006). Development and validation of the AmpFlSTR Yfiler PCR Amplification Kit: a male specific, single amplification 17 Y-STR multiplex system. J Forensic Sci.

[4] Davis C., Ge J., Sprecher C., Chidambaram A., Thompson J., Ewing M. (2013). Prototype PowerPlex Y23 System: a concordance study. Forensic Sci Int Genet.

[5] Planz J.V., Sannes-Lowery K.A., Duncan D.D., Manalili S., Budowle B., Chakraborty R. (2012). Automated analysis of sequence polymorphism in STR alleles by PCR and direct electrospray ionization mass spectrometry. Forensic Sci Int Genet.

[6] Pitterl F., Schmidt K., Huber G., Zimmermann B., Delport R., Amory S. (2010). Increasing the discrimination power of forensic STR testing by employing high-performance mass spectrometry, as illustrated in indigenous South African and Central Asian populations. Int J Legal Med.

[7] Geppert M., Edelmann J., Lessig R. (2009). The Y-chromosomal STRs DYS481, DYS570, DYS576, and DYS643. Leg Med.

[8] D’Amato M.E., Ehrenreich L., Cloete K., Benjeddou M., Davison S. (2010). Characterization of the highly discriminatory loci DYS449, DYS481, DYS518, DYS612, DYS626, DYS644 and DYS710. Forensic Sci Int Genet.

[9] Zeng X., King J.L., Stoljarova M., Warshauer D.H., LaRue B.L., Sajantila A. (2015). High sensitivity multiplex short tandem repeat loci analyses with massively parallel sequencing. Forensic Sci Int Genet.

[10] Churchill J.D., Chang J., Ge J., Rajagopalan N., Wootton S.C., Chang C.W. (2015). Blind study evaluation illustrates utility of the Ion PGM system for use in human identity DNA typing. Croat Med J.

[11] Olofsson J., Andersen M.M., Mogensen H.S., Eriksen P.S., Morling N. (2012). Sequence variants of allele 22 and 23 of DYS635 causing different stutter rates. Forensic Sci Int Genet.

[12] Myers N.M., Ritchie K.H., Lin A.A., Hughes R.H., Woodward S.R., Underhill P.A. (2009). Y-chromosome short tandem repeat intermediate variant alleles DYS392.2, DYS449.2, and DYS385.2 delineate new phylogenetic substructure in human Y-chromosome haplogroup tree. Croat Med J.

[13] Myers N.M., Ekins J.E., Lin A.A., Cavalli-Sforza L.L., Woodward S.R., Underhill P.A. (2007). Y-chromosome short tandem repeat DYS458.2 non-consensus alleles occur independently in both binary haplogroups J1–M267 and R1b3-M405. Croat Med J.

[14] Pumpernik D., Oblak B., Borstnik B. (2008). Replication slippage versus point mutation rates in short tandem repeats of the human genome. Mol Genet Genomics.

[15] Ballantyne K.N., Goedbloed M., Fang R., Schaap O., Lao O., Wollstein A. (2010). Mutability of Y-chromosomal microsatellites: rates, characteristics, molecular bases, and forensic implications. Am J Hum Genet.

[16] Ge J., Budowle B., Aranda X.G., Planz J.V., Eisenberg A.J., Chakraborty R. (2009). Mutation rates at Y chromosome short tandem repeats in Texas populations. Forensic Sci Int Genet.

[17] Adey A., Morrison H.G., Asan Xun X., Kitzman J.O., Turner E.H. (2010). Rapid, low-input, low-bias construction of shotgun fragment libraries by high-density *in vitro* transposition. Genome Biol.

[18] Warshauer D.H., King J.L., Budowle B. (2015). STRait Razor v2.0: the improved STR Allele Identification Tool – Razor. Forensic Sci Int Genet.

[19] Butler J.M., Decker A.E., Vallone P.M., Kline M.C. (2006). Allele frequencies for 27 Y-STR loci with U.S. Caucasian, African American, and Hispanic samples. Forensic Sci Int.

